# Generating interaction gestures in dyadic conversations using a diffusion model

**DOI:** 10.1371/journal.pone.0339579

**Published:** 2025-12-30

**Authors:** Yuya Okadome, Yazan Alkatshah, Yutaka Nakamura

**Affiliations:** 1 Faculty of Engineering, Tokyo University of Science, Katsushika, Tokyo, Japan; 2 RIKEN Information R&D and Strategy Headquarters, Sorakugun, Kyoto, Japan; 3 Hiroshi Ishiguro Laboratories, Advanced Telecommunications Research Institute International, Sorakugun, Kyoto, Japan; University of Houston, UNITED STATES OF AMERICA

## Abstract

As expectations for computer graphic (CG) avatars and conversational robots increase, enhancing dialogue skills via multimodal channels is crucial for achieving fluent interactions with humans. Thus, automatic interaction motion generation is essential for autonomous conversation systems. Natural motion generation, such as appropriate nodding, requires considering the behavior and voice of the conversation partner. However, current models generate motion from audio or text, neglecting interaction factors. In this study, we implemented an interaction diffusion model (IDM) that uses a diffusion approach and masking features to generate interaction behaviors for dyadic conversation. IDM accounts for two participants, using masks to generate features from conditional inputs. This allows for accommodating conditions like missing features and forecasting without retraining. The experimental results suggests that the model generates the human-like behaviors during conversation in 30 ms.

## Introduction

Motion generation for interaction scenarios is a crucial challenge associated with the advancement of natural communication robots [1]. Conversation behaviors encompass actions like nodding during speech and utilizing hand gestures to elucidate concepts like illustrating the size of an object. Additionally, facial features like facial action units, gaze, and head rotations [2] play important roles in natural and fluent communication [3,4]. However, the seamless fluidity observed in human-to-human conversations is not achieved by current dialogue systems, because many such systems are designed to respond solely to specific situations.

Conversation is inherently a “full duplex” scenario [4,5], and development of a motion generation model tailored for such situations is anticipated to enhance communication agents across various environments. In spoken dialogue systems, the full-duplex approach [5] for natural conversation focuses on effectiveness of fluent interaction. For example, video conference systems include large delays and a lack of presence, and there are differences between remote systems and fluent conversations like face-to-face conversations. Bidirectional nature must be considered when modeling interaction behavior.

In such scenarios, information of participants who attend the conversation should be processed simultaneously for effective real-time modeling behaviors during a conversation. Various methods for automatic motion generation during conversation have been proposed [6,7]. Developed systems include an attentive listening system where behaviors are determined by the partner’s actions or vocal input [8,9], and a lip-sync system utilizing audio data [10], both of which have undergone evaluation. Nonetheless, a limited number of systems exist that are capable of generating interaction behaviors during conversation.

For a data-driven approach, a deep generative model has been utilized for human behavior generation [11,12]. Generative models, such as variational autoencoders [13] and generative adversarial nets [14], have made great progress in the field of image generation. In particular, diffusion models [15,16] have attracted attention for many applications because the generation quality of these models is quite high. Motion-generation methods based on these generative models and text and audio features have been developed [17,18]. These methods are for motion generation in a round-by-round fashion, and a full-duplex situation [5] is not considered.

In this study, we aim to model and analyze interaction behaviors that consider a full-duplex situation, and investigate the availability of a generative model for the behaviors of two people in a dyadic conversation. In our previous research, we modeled and evaluated the behavior of a gesture consultant using a diffusion model [19]. One of the limitations in our previous studies was the insufficient evaluation of generated behaviors. In this paper, we adjusted the loss function to generate smoother motion, and, the feasibility of a generative model is investigated by conducting a subjective evaluation. The experimental results show that IDM can generate human-like gestures in practical computational time, and the model generates gestures exploiting the conversation partner’s behavior. Therefore, it is suggested that the generative model is applicable to automatically generating the interaction behaviors for an on-the-fly conversational system.

## Related work

In this section, we review related works on generative models for motion generation. Ongoing studies are exploring the use of deep generative models for generating human motion [6,7,11,12,17,18]. Techniques like variational autoencoders (VAE) and diffusion approaches are commonly employed in this area.

Li et al. [6] developed a method for gesture generation that incorporates audio information using a VAE. Yazdian et al. [7] proposed a generation method for various behaviors, and Guo et al. [20] and Zhang et al. [11] developed motion generation methods with text prompting. These models are based on vector quantized VAE [21], which learns pose sequences.

The quality of generated motions has been enhanced through use of diffusion approach [17,18,22]. Tevet et al. [17] and Zhang et al. [22] developed a motion generation method that relies on text information, successfully producing variety of motions. Alexanderson et al. [18] created a method that utilizes audio signals, enabling the generation of large and rapid motions, such as dance. These approaches are grounded in the denoising diffusion probabilistic model (DDPM) [15]. Despite high quality of generated motions, these methods only produce behavior of a single individual. These approaches receive text and speech information as “prompts,” and one person’s behavior is generated in a round-by-round fashion.

In this study, the full-duplex interaction behavior was modeled using a diffusion approach. However, the computational cost of DDPM is quite high due to large number of iterative calculations, making it unsuitable for online tasks [17]. To address this issue and reduce computational costs, the denoising diffusion implicit model (DDIM) [16] was proposed.

### Principles of diffusion models

Diffusion models such as DDPM and DDIM simulates a process in which original data *x*_0_ is progressively noised for *K* steps *x*_1:*K*_ according to $q(x_{k}|x_{0})$. In generative process, *x*_0_ is generated from samples drawn from $x_{K}\sim 𝒩(0,I)$.

#### Forward process.

The forward process describes the systematic corruption of initial data, *x*_0_, through the iterative addition of Gaussian noise across K discrete time steps, yielding a sequence of increasingly noisy data representations, *x*_1:*K*_. This process is characterized by the conditional probability distribution $q(x_{k}|x_{0})$, which quantifies the transformation from *x*_0_ to *x*_*k*_. In the context of DDIM, the noise magnitude $\sigma_{k}$ within the transition probability

$$q(x_{k-1}|x_{k},x_{0})=𝒩(\sqrt{\alpha_{k-1}}x_{0}+\sqrt{1-\alpha_{k-1}-\sigma_{k}^{2}}\frac{x_{k}-\sqrt{\alpha_{k-1}}x_{0}}{\sqrt{1-\alpha_{k}}},\sigma_{k}^{2}I).$$ (1)

is explicitly set to zero, where *x*_*k*−1_ is calculated by a deterministic procedure: the average of the normal distribution of the $q(x_{k-1}|x_{k},x_{0})$ is substituted. $\alpha_{k}\in [0,1)$ is the constant-decreasing sequence. To obtain *x*_*k*_, the reparameterization trick [13] enables the direct computation of *x*_*k*_ from *x*_0_ and a standard Gaussian noise ϵ, expressed as

$$x_{k}=\sqrt{\alpha_{k}}x_{0}+\sqrt{\left(1-\alpha_{k}\right)}\epsilon .$$ (2)

The value of *x*_*k*_ is dominated by the value of *x*_0_ since $\alpha_{k}$ is close to one when *k*, the step index, is small. Because the sequence $\alpha_{k}$ is designed to be monotonically decreasing in *k* and to approach zero, the state *x*_*k*_ gradually deviates from the original sample and asymptotically approaches a Gaussian distribution in the forward process.

#### Denoising process.

The denoising process constitutes the generative phase, wherein the primary objective is the reconstruction of the original data *x*_0_ from pure Gaussian noise $x_{K}\sim 𝒩(0,I)$. This is achieved through an iterative denoising procedure spanning *K* steps. In DDIM, {1,...,s,...S} which is the subsequent iteration step {1,...,K} is allowed to reduce the number of iterations in generative process.

The learned denoising process, denoted $p_{\theta }(x_{s-1}|x_{s})$ and parameterized by θ, aims to progressively infer the less noisy state *x*_*s*−1_ from the current noisy state *x*_*s*_. Within the IDM framework, ${\hat{x}}_{0}$ is directly estimated by $f_{\theta }$ from the noisy input *x*_*s*_. For steps where *s* > 1, *x*_*s*−1_ is computed based on this estimated *x*_0_ and *x*_*s*_,

$$x_{s-1}=\sqrt{\alpha_{s-1}}f_{\theta }(x_{s})+\sqrt{1-\alpha_{s-1}}\frac{x_{s}-\sqrt{\alpha_{s-1}}f_{\theta }(x_{s})}{\sqrt{1-\alpha_{s}}}.$$ (3)

The number of iterations for the denoising process in the DDIM framework is shorter than the process in DDPM because of using the subsequent iteration step. Therefore, by iteratively applying Eq (3) starting from a variable *x*_*S*_ sampled from a Gaussian distribution, as the index *s* runs from *S* to 0, *x*_*s*_ approaches to the high density regions of the original (training) samples. The function $f_{\theta }$, being nonlinear and local, determines the direction of the move like the gradient direction in a potential field. A large step size in this denoising process can introduce perturbations, potentially degrading the quality of the converged data.

## Materials and methods

**Fig 1:**
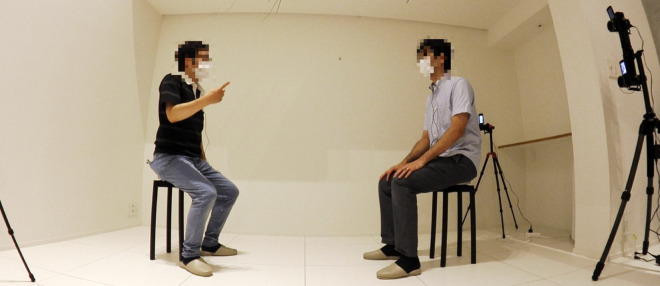
Data-recording environment. Two people sat on chairs placed at center position.

In this study, we used extracted features from videos during conversation for a generative model. The conversation data were recorded in the environment shown in Fig 1. The conversational data used in this study were collected as follows:

- Two people sit opposite each other,
- Video cameras are put behind each participant,
- Wireless microphones are put on each participant,
- For synchronization of each camera, the recording starts with the external trigger,

Note that both people sat during conversation session. We modeled the above types of dyadic conversation utilizing a generative model.

**Fig 2:**
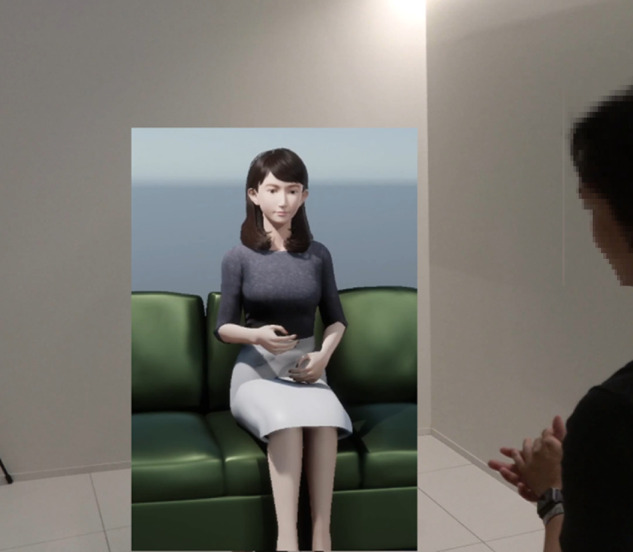
The snapshot of a video used in subjective evaluation.

In our study, we conducted an impression evaluation experiment to assess the quality of the generated motion. The recruitment period is from October 21/2024, to October 22/2024. Participants viewed short video clips created from the model’s output (see Fig 2) and evaluated their impressions (details in Results section). We commissioned an agency to conduct this survey, obtaining data from fifty evaluators (average age 41.3±7.3 years, ranging from 20s to 50s; 62% male, 38% female). The survey data, such as age and gender, were based on participants’ self-reported information. The data we received from the agency was already anonymized. The agency also informed the evaluators that their data would be anonymized and used exclusively for this research. This consent information was shown on the evaluators’ display at first, and only evaluators who agree participated in the experiment. The consent information was saved in CSV format. We confirmed with the relevant department at Tokyo University of Science that this procedure is acceptable.

### Definition of features

Conversations between one gesture consultant and three individuals were recorded, and two 15-minute sessions were conducted for each individual. As a result, the total amount of video was approximately 1.5 h.

**Fig 3:**
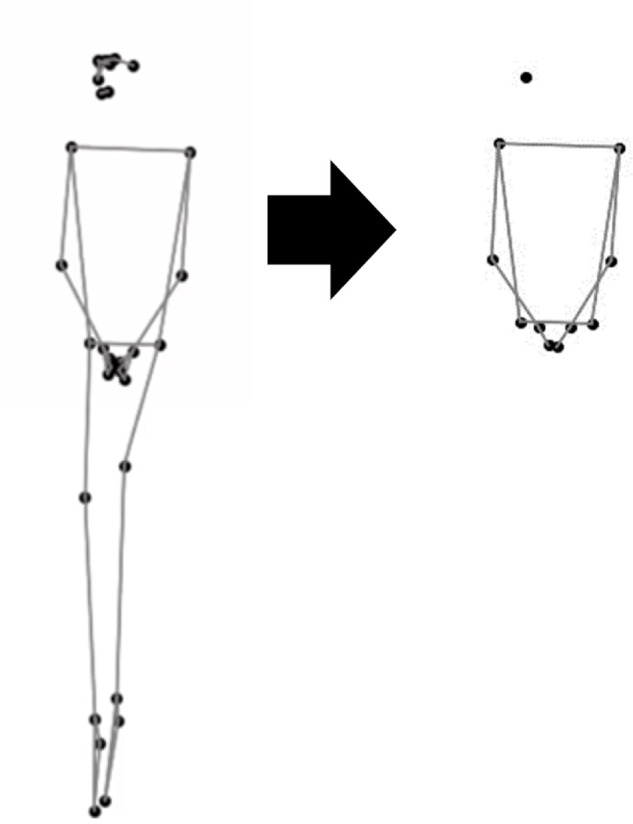
Feature points extraction by an image based motion capture system.

MediaPipe Pose Landmarker (https://ai.google.dev/edge/mediapipe/solutions/vision/pose_landmarker) is used to extract pose information at 30 fps. Then, we extracted eleven feature points: nose, both shoulders, both elbows, both wrists, both pointing fingers, and left and right waists (Fig 3) because participants were sitting during the conversation, and the lower limbs empirically did not move much. A low-pass filter is applied to a sequence of positions of eleven points, and the sequence is down-sampled to 5 fps by sampling at the same time interval.

The speech signal is obtained from a wireless microphone attached to each participant. The sampling rate of speech signal is 48 KHz. For the obtained speech signal *A*(*t*), the features using values measured in last 0.2 s, were computed by $\hat{A}(t)=\max_{t\in (t-0.2,t]}A(t{)}^{2}$. Thus, the speech signal was down-sampled to 5 Hz to calculate maximum power for past 48,000/5=9,600. The dataset for tasks was constructed by concatenating poses and sound strength after downsampling.

**Fig 4:**
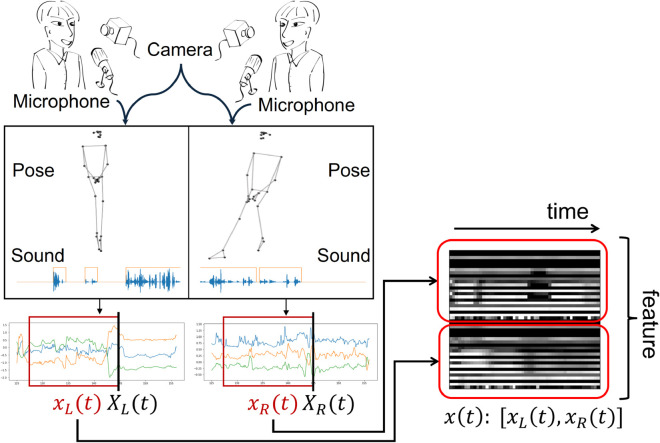
Relationship between X_*_(*t*), x_*_(*t*), and x(t). X_*_(*t*) is feature at a specific time, and x_*_(*t*) is *T* time-step features for each participant. *x*(*t*) is the concatenated features of *x*_*L*_(*t*) and *x*_*R*_(*t*).

In this study, a motion generation model for interaction behavior during dyadic conversation was considered. Because the interaction data include social information, past behavioral features must be used. *T* time-step features of the two participants are defined as $x_{L}(t)=[X_{L}(t-i)|i=0,...,T],x_{R}(t)=[X_{R}(t-i)|i=0,...,T]$, where *X*(*t*) is the feature at a specific time. Fig 4 shows the relationship between *x*(*t*) and *X*(*t*) at time *t*. The time indices of features $\cdot_{L},\cdot_{R}$ are consistent.

To generate interaction behaviors, the features of two people must be handled simultaneously. The feature at *t* is defined as $x(t)=[x_{L}(t),x_{R}(t)]$. Hence, *x*(*t*) is a concatenated feature of the two terms. In the proposed method, the generative model for *x*(*t*) is considered.

### Representation of time-series data

In the proposed method, part of two people’s motion *x*(*t*) (as discussed in section Definition of features) is “masked,” and the feature in the masked region is generated by a diffusion approach. The original data from training data in diffusion approach are denoted as *x*_0_(*t*). The mask shape for multidimensional time-series data was also considered because the shape of the mask affects performance of motion generation.

**Fig 5:**
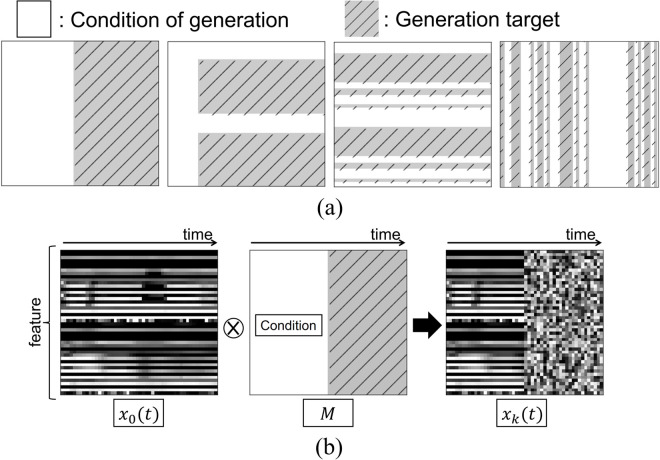
Mask shapes used in this paper. Horizontal and vertical axes show timestep and features. (a) Mask patterns used in the experiment. White and shaded regions indicate regions with values 1 and 0, respectively. (b) Examples of *M*, *x*_0_(*t*), and *x*_*k*_(*t*). Features in unmasked region are noised, and hence, right region of *x*_*k*_(*t*) is noised. Generation target of IDM is noised region of *x*_*k*_(*t*).

Fig 5(a) shows examples of mask shapes. The mask *M* is represented as the tensor $M\in \{0,1{\}}^{L\times T}$ where *L* and *T* are the number of features and the time length. The size of *M* is the same as *x*_0_(*t*), and *M* controls whether the input features are missing. The values of the variables in the region where the value of the element of *M* is 1 are fixed. (In the following, we use *M* = 1 to indicate the region in which *M* has elements equal to 1.) Conversely, the variables in the region (1−M)=1 are updated, and they are the target variables to be generated by the diffusion model. **1** is the L×T matrix of all ones. The operation is achieved by element-wise multiplying *x*_0_(*t*) with the mask, *i.e.,* performing the Hadamard product.

The training utilizes masks that reflect actual control situation, including missing frames, always unavailable sensors, unavailable sensors for some time, and observations only up to current time step to empirically improve generalization ability. The masks utilized in the proposed method are designed to simulate scenarios that happen during the actual operation of the system. For instance, scenarios include unknown future states for motion generation, or frame loss due to network latency. Therefore, by augmenting the data with these real-world situations through the use of masks, we expect an enhancement in the model’s generalization ability and handling of multiple tasks. For example, *M* is used to delete all features after a certain time step or features that are missing some modalities, such as the right hand and voice.

## Interaction diffusion model

Because each person affects the other in an interaction setting, a motion generation model for one person that generates gestures from one’s voice [6,18] is not sufficient. For instance, nodding and smiling [4] are expressed as reactions for conversation partners; hence, instantaneity of these reactions should be considered. To solve this problem, our proposed model, viz., IDM, models two people’s behaviors as a joint probability.

### Forward process with masked time-series data

Noised data *x*_*k*_(*t*) at iteration time-step *k* in forward process are calculated using *x*_0_(*t*) and *M*:

$$x_{k}(t)=M⊗x_{0}(t)+(1-M)⊗\left(\sqrt{\alpha_{k}}x_{0}(t)+\sqrt{\left(1-\alpha_{k}\right)}\epsilon \right)$$ (4)

where ⊗ is the Hadamard product. Fig 5(b) shows the relationship between *x*_0_(*t*),*M*, and *x*_*k*_(*t*). For Eq (4), noise is added only to the region, (1−M)=1, in the forward process. However, generative model can refer to the data history by retaining information on the region, *M* = 1. The model trained with these masks can generate future series while maintaining consistency with past series.

The parameters θ of the function $f_{\theta }(x_{k})$ in the proposed IDM were trained using simplified loss function [15,16]. The added noise ϵ of $x_{0}=(x_{k}\;-\;\sqrt{1-\alpha_{k}}\epsilon )/\sqrt{\alpha_{k}}$ is estimated using the DDIM. In the IDM, *x*_0_ is directly estimated by $f_{\theta }$, that is $f_{\theta }(x_{k},k)=\hat{x_{0}}$, which is similar to the approach of Ramesh et al. [23]. The loss function with mask $L(x_{0},{\hat{x}}_{0},M)$ is calculated as the L1 loss:


$$L(x_{0}(t),{\hat{x}}_{0}(t),M)=||(1-M)⊗(x_{0}(t)-{\hat{x}}_{0}(t))|{|}_{1}$$

$$+||(1-M)⊗(\Delta x_{0}(t)-\Delta {\hat{x}}_{0}(t))|{|}_{1},$$ (5)

where Δx(t)=x(t)−x(t−1) is the one-step difference feature of behavior. Because the target of our study was a dyadic conversation, physical constraints, for example, the maximum acceleration in human motion, can be added to the error function. The design of loss function will be the subject of future research.

### Denoising process of IDM

As mentioned in section II-Ab, {1,...,s,...S} which is the subset of {1,...,K} is used in the generative process. In the IDM, features in the region $\left(1-M)⊗x_{s}(t\right)$ are generated. Hence, $M⊗x_{0}(t)$ is handled as known information.

The progressive denoised features *x*_*s*−1_(*t*) with *M* becomes

$$x_{s-1}(t)=M⊗x_{0}(t)+(1-M)⊗{\bar{x}}_{s-1}(t),$$ (6)

and, from Eq (3),

$${\bar{x}}_{s-1}(t)=\sqrt{\alpha_{s-1}}f_{\theta }(x_{s})+\sqrt{1-\alpha_{s-1}}\frac{x_{s}-\sqrt{\alpha_{s-1}}f_{\theta }(x_{s})}{\sqrt{1-\alpha_{s}}}.$$ (7)

As discussed in the previous section, *x*_0_(*t*) is directly estimated for each iteration time step by using $f_{\theta }$. By repeating this procedure until *s* = 1, interaction behaviors are generated.

### Network architecture

**Fig 6:**
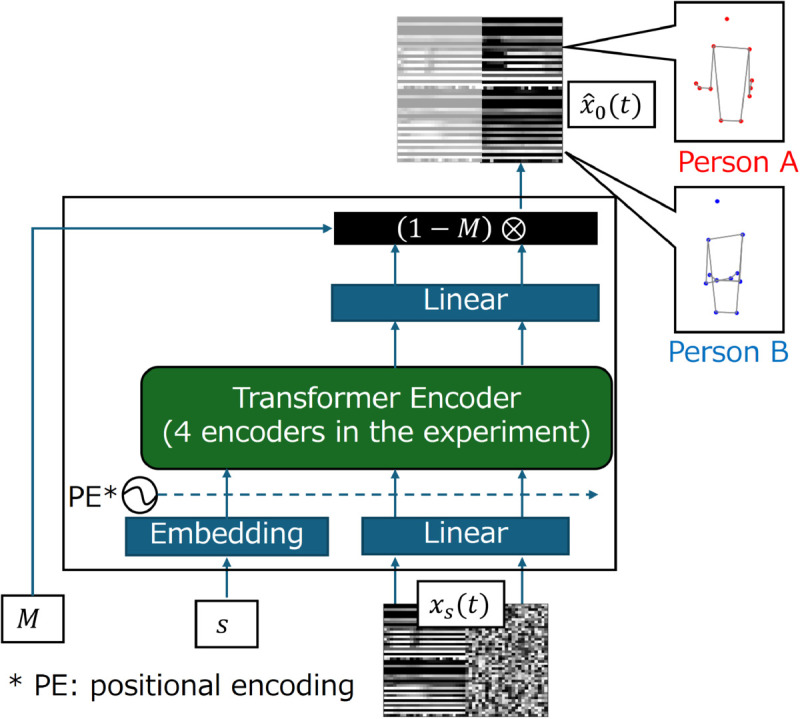
IDM network architecture. IDM receives M, s, and $x_{s}\left(t\right)$, and output is estimated result of $x_{0}$. Two people during dyadic conversation are included in *x*_0_(*t*), and both participants’ behaviors are always generated by IDM.

Fig 6 shows the IDM network architecture. The IDM receives *x*_*s*_(*t*), iteration time-step *s*, and *M*, and then the generation result of the masked region $(1\;-\;M)⊗{\hat{x}}_{0}$ is output. Note that the features in the region *M* = 0 are ignored, and the shape of $(1-M)⊗{\hat{x}}_{0}$ is identical to that of *x*_0_(*t*). The motions of the behaviors of the two people are included in the results.

The embedding layer is used for converting *s* to 256-dimensional features, and the noised signal *x*_*s*_(*t*) is also converted to 256-dimensional features by a full connection layer. These converted features are concatenated, and the ordering information is added by positional encoding. In our model, a four-stacked transformer encoder [24] model is used as the network architecture because the unmasked region $M⊗x_{0}(t)$ can be utilized for any time in the data. The output of the transformer is input to a fully connected layer, and the number of feature dimensions is matched with *x*_0_(*t*). The codes of IDM will be available at: https://github.com/animawer/idm/tree/main.

## Results

IDM was applied to motion generation for a dyadic conversation, and generation results were verified. In this experiment, we constructed a training dataset from recorded video of dyadic conversations. To ensure consistency, one individual was a skilled gesture consultant with experience in choreography instruction and proficient gesture expression (The person leads the gesture consulting company (KiQ: https://kiq.ne.jp/en).). A computer with an NVIDIA Quadro A5000 graphics card was used for calculations.

### Training settings

The IDM was trained on the dataset and applied to motion generation task. Two-minute samples from one session were divided into test data. The time length of data *T* was empirically set to *T* = 50, that is, 10 s clips were extracted. The total sample size (number of clips) for training was 21,597. The number of iteration time steps in generative process was set to 10. The Adam optimizer was used to train the network, and the learning rate was set to $1.0\times 10^{-4}$.

### Numerical evaluation of generated samples

**Fig 7:**
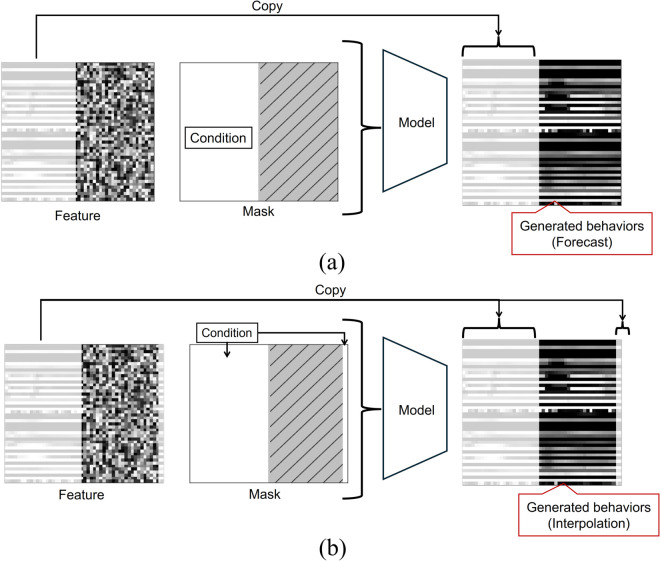
Mask shapes for each condition. (a) forecast and (b) interpolation.

The IDM can be used to generate samples in various conditions without retraining by changing the shape of the masks. In this experiment, two motion generation tasks were conducted: forecast and interpolation. In the forecast condition (Fig 7(a)), observations of both participants’ features at the current time are considered, and future behaviors of both people are generated simultaneously. In the interpolation condition (Fig 7(b)), features at the current time and defined terminal states of both people are considered, and behaviors of both people between current states and terminal states are interpolated.

The proposed method was also compared with a variational autoencoder with arbitrary conditioning (VAEAC) [25], which can process masked features, DDPM with MASK (DDPM), which has the same network structure and optimizer as IDM (DDIM-based), and IDM without velocity term during training (IDM*). In the DDPM, the training target is the noise, similar to the original DDPM [15].

Following this section, we named each model as VAEAC_*F*_, DDPM_*F*_, IDM*_F_ and IDM_*F*_ for the forecast task, and VAEAC_*I*_, DDPM_*I*_, IDM*_I_, and IDM_*I*_ for the interpolation task. Note that 25-step motions were generated in this experiment.

The task overview and mask used for the forecast are shown in Fig 7(a). From the mask shape, the generation target includes all the features after *T*/2 = 25. This setting is a typical case of robot/agent motion generation, in which observations are obtained up to the current time.

The task overview and mask shape for the interpolation are shown in Fig 7(b). In this setting, the final state of the behavior is given, and intermediate motions are generated. The generated motions were investigated to connect them to the terminal pose.

#### Experimental results

Fréchet’s inception distance (FID) [17,26] and multimodality [17,27] were calculated for each condition, and the generated motions were evaluated. For FID, the generated gestures were compared with the consultant’s gesture. A smaller FID value means that the generated results and professional behavior are similar.

Multimodality is a criterion that determines the diversity of the motions generated for certain clip data, and features with large movements are crucial for the criterion. In this experiment, multimodality was calculated using the nose and right and left wrist features because arms move largely during a conversation for gestures. Additionally, participants are seated and in-person, resulting in small waist movement. Consequently, the features are excluded from the multimodality calculations. FID and multimodality were calculated on the generated results for the test data.

**Fig 8:**
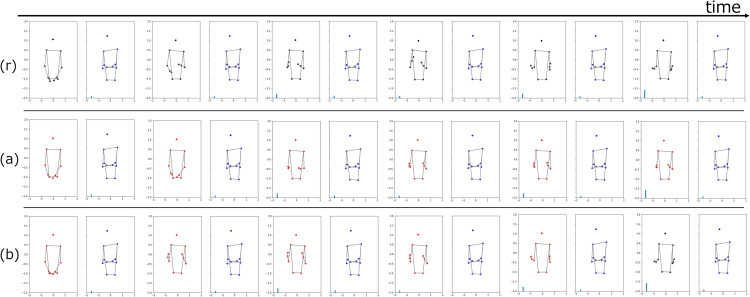
Generation results of forecast and interpolation conditions by IDM. Black, blue, and red shapes indicate original consultant, conversation partner, and generated poses, respectively. (r) Original signal, (a) generated result of forecast, and (b) generated result of interpolation.

Fig 8 shows the motion generation results of the IDM. Gestures during conversation were generated under all conditions (forecast and interpolation). The pose variation of the generated motions was smaller than that of the original data (Fig 8(r)). For the generated results, the gestures of interpolation (Fig 8(b)) tended to be larger than those in the forecast condition (Fig 8(a)).

The scores of the generation results by each model are listed in Table 1. Because VAEAC is not an iterative process, the computational time is shorter than that of IDM; however, its FID and multimodality scores are not higher than those of IDM. From the results of the multimodality of VAEAC, the variety of generated motion is small, and hence, it is considered that FID is also not improved.

**Table 1:** FID, multimodality, and computational time for each condition.

Forecast task			
Method	FID↓	Multimodality↑	Comp. Time[ms]
VAEAC_*F*_	1.84 ± 0.01	$5.4\times 10^{-3}$ ± $5.3\times 10^{-3}$	**5.9**
DDPM_*F*_	**0.51 ± 0.13**	0.16 ± 0.01	529
IDM_*F*_	0.54 ± 0.11	0.18 ± 0.01	28
IDM*_F_ [19]	0.88 ± 0.09	**0.24 ± 0.15**	28
Interpolation task			
Method	FID↓	Multimodality↑	Comp. Time[ms]
VAEAC_*I*_	1.86 ± 0.01	$5.0\times 10^{-3}$ ± $5.1\times 10^{-3}$	**5.9**
DDPM_*I*_	0.44 ± 0.16	0.11 ± 0.01	529
IDM_*I*_	**0.41 ± 0.13**	**0.13 ± 0.01**	28

Best results for each criterion are highlighted. Mean and standard deviation are calculated from 10 learned models. Symbol * means “same as above.” IDM* means IDM without velocity term. Note that FID for the gesture of individuals is 4.97.

DDPM_*F*_ has a higher computational cost as expected, while the quality (FID) is similar to IDM_*F*_. As an inherent property of the DDIM, the iterations are significantly reduced. When compared to IDM_*I*_, the improvement in FID for DDPM_*I*_ is marginal. Physical connections of DDPM at the endpoint are less affected since DDPM estimates the noise rather than the original signal. Because the computational time required for IDM is approximately 30 ms, which is significantly shorter than the 530 ms required for DDPM, IDM is expected to be well-suited for online motion generation. From the results between tasks, by changing the shape of the mask, the tendency of the generation results also changes.

For the comparison between IDM*_F_ [19] and IDM_*F*_, the FID of IDM_*F*_ is lower than that of IDM*_F_. This means that the velocity term works for modeling human behavior. However, the multimodality of IDM_*F*_ is also lower than that of IDM*_F_. Because the physical constraint is added during training IDM, the variety of behavior is reduced.

#### Longer-term motion generation.

**Fig 9:**
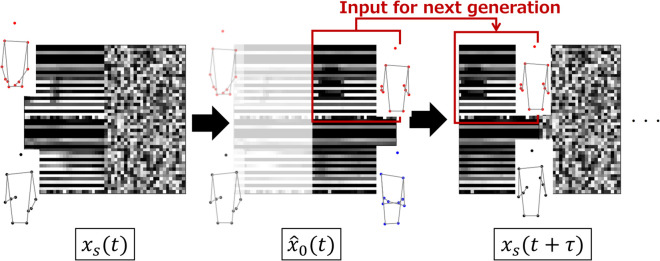
Auto-regressive input features. Generated consultant’s gesture is used as input for successive generation.

In this experiment, a longer motion, which assumes the existence of a conversation partner, was generated. Motions were auto-regressively generated by inputting the generated consultant gestures into the next motion generation. Fig 9 shows the input of the next time $x_{t+\tau }$ with the generated gesture of the consultant at time *t*. When the generation process begins, the features of the conversation partner are assumed to be observed. The unit of generated motion was 25 steps, and 100 steps of motion (20 s motion) were generated.

In the longer-motion generation task, diversity [6,17] was used for the evaluation. Diversity is an evaluation criterion for the number of different motions in a long motion. The generated long motion was divided into non-overlapping clips, and the differences between the clips were evaluated. Diversity was also calculated using nose and right and left wrist features. In addition to diversity, FID for the last clip was also evaluated to investigate the effect of long-term generation.

Table 2 shows the results of the VAEAC, DDPM, and IDM. The diversity of three generative models is lower than the consultant’s motion, that is, the variety of motions of the generative models is smaller than the original. Because the score for IDM is higher than that for VAEAC and DDPM, various behaviors between clips are generated by IDM. The standard deviation of the scores of IDM is larger than that of DDPM. Because the number of iterations in the IDM is smaller than that of DDPM, the output behaviors tend to fluctuate.

**Table 2:** Diversity and FID for each condition.

Method	Diversity↑	FID↓
Consultant	31.91	-
VAEAC	13.74 ± 9.34	6.69 ± 0.042
DDPM	22.12 ± 0.97	1.81 ± 0.95
IDM	**24.40** ± **2.12**	**1.28** ± **0.59**

Mean and standard deviation are calculated from 10 learned models. “Consultant” refers to the original consultant’s behaviors.

For the FID, the difference in the score of VAEAC is much larger than that in the previous experiment. The FID of IDM and DDPM was about two and three times larger than in the previous experiment. Because the actual consultant’s features are not used as the condition, the FID worsens according to the length of the behavior. Maintaining consultant-like behavior for long-term generations is one of our future research directions.

### Qualitative evaluation based on human impression assessment

**Fig 10:**
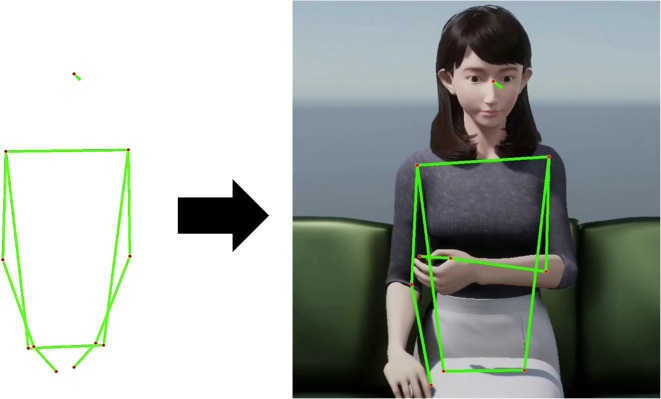
Example of motion transformation from pose to CG-agent.

This experiment evaluated the relationship between the generated and human behavior during conversation using a CG-agent that imitates the android robot ERICA (Fig 10) [28]. Although the results of previous experiments suggest that IDM can generate human-like behavior, the naturalness of generated behavior during communication is not evaluated.

Participants were advertised on a cloud platform through an agency that handled contracts and operations. The number of participants was fifty (age 41.7±7.3, 31 males and 19 females).

#### Experimental settings.

**Fig 11:**
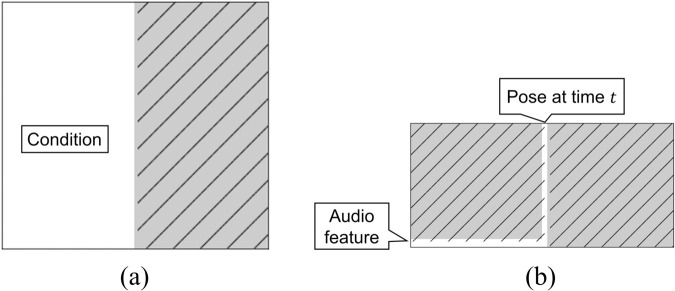
Mask shape for each condition. (a) the mask for the IDM condition, and (b) the mask for the speech condition.

In this experiment, we compare three methods: 1. copy of human behavior (COPY), 2. gestures generated from conversation voice (SPEECH), and 3. IDM forecast (IDM) which the mask shape is shown in Fig 11(a). COPY extracts the behavior from the original conversation video and is considered the best full-duplex model. SPEECH uses only the voice information of the consultant for IDM during training and generation by a specific mask structure (Fig 11(b)), and this model is considered the half-duplex model. Note that to preserve the connection between the current and previous generated behavior, the mask is designed so that the model can refer to the position information at time *t* (current time index). Because the FID of the SPEECH is 0.54±0.15, the performance of SPEECH is close to the IDM_*F*_. The last model is the IDM, which is the same model as in the previous experiment. The performance of generated behaviors of IDM is evaluated by comparing IDM, half-duplex, and best full-duplex model.

These copied and generated behaviors are used to control the ERICA-like CG agent [28]. In this experiment, our aim was to investigate the differences in human and generated behaviors and the effect of considering other people’s behavior for communication gesture generation.

For this experiment, six dyadic conversation videos were prepared, and the time length of each video was 20 s. Generation conditions such as SPEECH and IDM generates five behaviors for each conversation video, *i.e.,* thirty videos are generated in total. Fifty participants evaluated motions of COPY, SPEECH, and IDM conditions. Five sets of videos are prepared, and for each set, there are 18 videos, which are six conversation videos for each of the three conditions. Videos in one set were presented to 10 participants. As a result, we obtained 300 data points for each condition.

The objective of this experiment is to investigate whether the generated and copied motions are preferable and natural for participants. To assess this, we utilized the Japanese version of the GodSpeed questionnaire [29], which evaluates five factors: anthropomorphism, animacy, likeability, perceived intelligence, and perceived safety. During the evaluation process, a participant was shown one video and subsequently completed the GodSpeed questionnaire. This procedure was conducted for a total of eighteen different videos, and we collected the evaluation results accordingly.

#### Experimental results.

**Fig 12:**
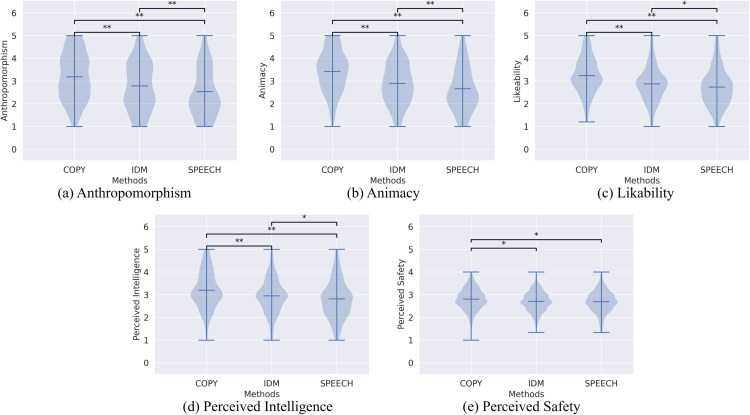
Results of human evaluation for cope and generated motions. Mean and results of Steel-Dwass test are illustrated. Horizontal and vertical axes are motion conditions and evaluation values, respectively. Characters “*” and “**” show significant differences with *p* < 0.05 and *p* < 0.01, respectively.

The following results present the results of the Steel-Dwass test for each aspect of the questionnaire. Fig 12 and Table 3 show summary of the human evaluation for each condition and their effect size between COPY, SPEECH, and IDM.

**Table 3:** The Cohen’s d values between COPY, SPEECH, and IDM results.

Godspeed factors	COPY v.s. IDM	COPY v.s. SPEECH	IDM v.s. SPEECH
Anthropomorphism	0.385	0.627	0.242
Animacy	0.568	0.808	0.249
Licability	0.456	0.618	0.175
Perceived intelligence	0.309	0.459	0.169
Perceived safety	0.214	0.229	0.026

There are significant differences between COPY, IDM, and SPEECH in terms of anthropomorphism, animacy, likeability, and perceived intelligence. COPY shows a higher evaluation value for all aspects of anthropomorphism, animacy, likeability, perceived intelligence, and perceived safety. The effect sizes between COPY v.s. IDM, COPY v.s. SPEECH are medium. The distributions of scores of IDM are at a lower position than those of COPY, but the value distributions are at a higher position than those of SPEECH, except for perceived safety.

For IDM and SPEECH, there are significant differences in terms of anthropomorphism and animacy. Although these effect sizes are small, the statistical test shows IDM can generate more human-like behavior than SPEECH. These results suggest that information from other participants during conversation is necessary for modeling natural interaction behavior. The impact of the model’s low accuracy, resulting from insufficient training data, may have made the differences in subjective evaluations less clear. Evaluating the model as the training data increases remains a future challenge.

## Discussion

From the results of FID (Table 1), the ascending order of scores is interpolation and forecast, and multimodality value of forecast is the highest. For interpolation, existence of a physical constraint as a terminal state limits the range of possible motion candidates. In contrast, during forecasting condition, the simultaneous generation of both consultant’s and partner’s behaviors leads to increased multimodality and FID values.

In long-term motion generation task described in section Longer-term motion generation, consultant’s generated gesture was input auto-regressively into the IDM. When the gesture of conversation partner was also included as input, the FID of final generated clip was 1.28, which was worse than the result shown in Table 2. If consultant’s behaviors are solely referenced by trained IDM, we expect the FID to be a similar value. These results also indicate that IDM processes information from both individuals simultaneously.

One of the limitations is the validity of the numerical evaluations. FID and other values indicate how close the generated motions are to the training samples, but it does not necessarily indicate the appropriateness of the generated samples. For example, a low FID sample may not be positively evaluated by the conversation partner as shown in the results of the subjective evaluation. Thus, we consider the evaluation experiment of controlling an actual robot or CG-avatar with the proposed method as the next step.

In the performance evaluation of this paper, only the behaviors of a single expert (gesture consultant) during conversation were used. Due to variations in movements stemming from individual differences, such as the personality or cultural backgrounds, evaluating performances for accounting each individual remains a challenge for the future. Recording large amounts of data from a single individual is impractical. Therefore, it is necessary to develop pre-training methods using datasets that are not limited to the target individual, such as well-known datasets of behaviors during conversations, and to verify their effectiveness.

Experimental results of human impression suggest that IDM exploits conversation partner’s information. Although the evaluation score, FID, of the motion generation model from speech is same as IDM, there are significant differences in human impressions. Note that the performance indexes used in this paper, *i.e.*, the FID and multimodality, are not designed to evaluate communication behavior for simplicity. That is, the temporal structures between multidimensional time series observed from two interacting individuals are not considered, although the relationship between the behavior of the pair engaged in dialogue would affect them [30]. From the above points, we implicitly show dependency of gesture expression during dyadic conversations.

From the subjective evaluations, it is suggested that the full-duplex model is better than that of the baseline (SPEECH) model. In this experiment, the motions are generated in an offline manner, and it is expected that a similar quality of motions can be generated in a real-time situation by the proposed model because the model generates behaviors in the computational time of 30 ms. This indicates that the proposed method is applicable for actual full-duplex situations.

In the experiment, we used the model trained on 1.5 h of data, and the sample size significantly impacts the performance of the trained model. To explore this issue, we compared the FID of a model trained on 1.5 hours of data with models trained on 0.75 hours and 4.0 hours of training data. Note that the 0.75 hours of data were obtained through sampling at the same time interval from the 1.5 hours of data, while the 4.0 hours of data represent the total amount of data we collected, including 2.5 hours of data from non-professional people. The FID values for the models trained with 0.75, 1.5, and 4.0 h of data are 0.88±0.71, 0.54±0.11, and 0.50±0.13, respectively. However, the differences are not substantial. Better FID does not mean that the generated gesture is actually close to the gesture of the model person, because the gesture similarity is not related to the loss function during training. Therefore, we conclude that using the model trained on 1.5 hours of data is appropriate for evaluations.

In this study, pose features are employed as modeling target because of their feasibility, because the pose modality is easily replicable by robots and CG avatars. While some nonverbal cues like facial expressions, gaze, and finger positions are crucial, they are not easy to obtain using general sensors like cameras and microphones. Additionally, based on our knowledge [30], if OpenFace is applied to Japanese participants and utilized for extracting facial expressions in real-time, extracted facial expression includes large noise and produces unstable results. Because it is difficult to distinguish whether the training result indicates the output property of OpenFace or actual facial expressions, we used pose information, which can be stably obtained. Combining facial expressions with IDM is our future project.

## Conclusion

In this study, we investigated the availability of the generative model for generating interaction behavior in dyadic conversation by numerical and subjective evaluations. The model utilizes diffusion approach, incorporating mask that applies to the features of both participants in conversation. IDM is designed to simultaneously generate the movements of two individuals, as generation is conditioned by the mask and characteristics of both participants. Furthermore, the DDIM framework is utilized to facilitate rapid motion generation.

IDM was utilized for the task of generating interaction motions, and its performance was assessed based on several criteria: FID, multimodality, diversity, computational time, and human impressions. The results of motion generation indicate that scores are “good” and the process is capable of producing fast calculations. Additionally, trends in the generation results were observed by altering the shape of the mask. The model effectively utilizes information from the conversation partner to generate interaction behaviors.

The poses of a human captured in video were generated by IDM. By utilizing these features, we expect to convert the poses into joint angles of a robot, enabling it to replicate the motions. Our future studies will focus on designing this conversion function to develop a communication robot that can generate motion online using IDM. Reproducing actions while considering individual differences, such as those exhibited by a skilled gesture consultant, and exploiting the generation results of a conversation partner also remain subjects for future research.
